# Effect of methanol leaf extract of *Dalbergia saxatilis* Hook.f (fabaceae) on renal function

**Published:** 2016

**Authors:** Fatima Ismail Hassan, Zezi Abdulkadir Umar, Danmalam Umar Habib, Yaro Abdullahi Hamza

**Affiliations:** 1 *Department of Pharmacology and Therapeutics, Ahmadu Bello University, Zaria, Nigeria*; 2 *Department of Pharmacognosy and Drug Development, Ahmadu Bello University, Zaria, Nigeria*; 3 *Department of Pharmacology, Bayero University, Kano, Nigeria*

**Keywords:** *Dalbergia saxatilis*, *Renal function*, *Histopathology*

## Abstract

**Objective:**

*Dalbegia saxatilis* (*D.saxatilis*) is used as a decoction in traditional medicine for ailments such as cough, small pox, skin lesions, bronchial ailments and toothache. This study is aimed at evaluating the toxic effect of methanol leaf extract of *D.saxatilis *on renal function.

**Materials and Methods:**

Wistar rats of both sexes were divided into four groups of five: control animals (group 1) received distilled water 1 ml/kg while groups 2, 3 and 4 were given graded doses of the extract (250, 500 and 1000 mg/kg body weight, respectively) daily for 28 days. Body weight changes were estimated by weighing the rats twice weekly using digital weighing balance. After 28 days, blood samples were obtained for evaluation of renal indices and the kidney was used for histopathology. Data were analysed using one–way and repeated measures ANOVA using SPSS version 20.

**Results:**

Significant weight increase in all groups were observed (p<0.05). Significant reduction in electrolytes concentration was observed following treatment with extract (250 and 500 mg/kg) (p<0.05). Histopathological findings of the kidney revealed massive necrosis of the glomerulus with tubular distortion and lymphocyte hyperplasia at 250 and 500 mg/kg while intense glomerular and tubular necrosis was observed at 1000 mg/kg of the extract.

**Conclusion:**

Since different doses of the extract caused reduction in electrolyte concentration and damage to the kidney it is suggested that prolonged administration of the extract is associated with increased risk of kidney toxicity*.*

## Introduction

Plants used in traditional medicine are relatively safe, but some may have undesirable adverse effects which may be due to over dosage or wrong usage and these may lead to toxicity and death (Okigbo et al., 2009 ▶). Toxicity studies are conducted to provide better understanding of the potential hazard of a test item and to estimate its safety margins. These safety margins are used to determine an initial safe starting dose for clinical trials, a safe dose for long-term use in humans through longer clinical trials, and ultimately to achieve successful review of registration dossiers to support marketing approval and use of new medicines by the wider population (Robbinson et al., 2009 ▶). The relationship between the dose of a toxicant and the response produced follows a predictable pattern, increase in the dose of a toxicant leads to increased response, which may be either in terms of the proportion of the population responding or in terms of the severity of the graded responses (Ballantyne et al., 1993 ▶). Different parts (leaves, bark, and roots) of *D.saxatilis* belonging to the familyfabaceae are used in traditional medicine for various ailments such as cough, small pox, skin lesions, bronchial ailments and toothache (Saha et al., 2013 ▶). This plant is native to Angola, Cameroon, Gabon, Ghana, Guinea, Guinea Bissau, Ivory Coast, Liberia, Nigeria, Senegal, Sierra Leone and Zaire (Saha et al., 2013 ▶). Various studies showed the repellant, insecticidal and antimicrobial effects of dried leaves of the plant (Okwute et al., 2009 ▶). Also, uterine contractile activity was shown to be due to the presence of a biologically active principle that act as a competitive inhibitor of β_2_ - selective adrenoreceptor agonist and enhanced α-adrenoreceptor function (Uchendu, 2000 ▶; Uchendu, 2003 ▶). Moreover, anticonvulsant activity, anxiolytic and muscle relaxant activities (Yemitan and Adeyemi, 2003 ▶; Yemitan and Adeyemi, 2005 ▶), as well as antioxidant activity were studied (Sofidiya et al., 2006 ▶). The effect of the plant on renal function has not been examined, thus the objective of the study was to evaluate the toxic effect of methanol leaf extract of *D.saxatilis* on renal function. 

## Materials and Methods

**Plant materials**

Fresh leaves of *D.saxatilis* were collected in June, 2013 at Galadimawa, Giwa Local Government Area, Kaduna State. The plant was identified, authenticated by a taxonomist in the Herbarium of the Department of Biological Sciences of Ahmadu Bello University, Zaria, and compared with a voucher specimen number 717 previously deposited as reference. The collected leaves were air-dried for two weeks and the size was reduced with mortar and pestle, then, extracted by cold maceration with 100% methanol for 48 hr with intermittent shaking, and filtered. The filtrate was evaporated using a water bath at 50°C to obtain a solid residue.

**Animals**

The protocol of the study was approved by the Departmental Animal Ethics Committee (DAC), Department of Pharmacology and Therapeutics, Faculty of Pharmaceutical Sciences, Ahmadu Bello University, Zaria (ABU) with approval number DAC/IW-OT/06-13. Wistar rats of both sexes weighing 100-150g were obtained from the Animal House facility of the Department of Pharmacology and Therapeutics. The animals were allowed free access to standard feed and water *ad libitum*. They were kept in clean plastic cages filled with saw dust which was replaced every three days. 

**Study design**

Wistar rats of both sexes were divided into four groups of five rats each: group 1, which served as control received distilled water 10 ml/kg while groups 2, 3 and 4 were given graded doses of the extract (250, 500 and 1000 mg/kg body weight), respectively daily for 28 days. The doses were selected based on median lethal dose. The highest dose is 20 %, intermediate is 10 % and the lowest dose is 5% of the LD_50_.

**Determination of average body weight**

Rats were weighed twice weekly and the average of change in weight was calculated using weighing balance (AE240 dual range, Mettler instrument corporation, USA).

**Renal indices**

Blood samples were collected from rats sacrificed on the 29^th^ day of the experiment into plain bottles, allowed to clot and centrifuged at 3500 rpm for 10 min. The sera were separated, stored at -4°C, and used for evaluation of renal indices, including serum urea nitrogen, creatinine, chloride, sodium, potassium, and bicarbonate using commercial kits obtained from Reckon Diagnostics P. Ltd, India.

**Histopathology**

The kidneys were removed and fixed in 10% formalin for at least 48 hr. They were then processed routinely, and the tissues were embedded in paraffin wax. Histological sections were cut at 5 μm and stained with routine haematoxylin and eosin. Afterwards, samples were examined by a consultant histopathologist. Lesions were observed and assessed. Photomicrographs of representative lesions were taken 250 x magnification.

**Statistical analysis**

Data were expressed as mean ± standard error of mean (SEM). Statistical differences between means of different groups were determined using one way and repeated measures analysis of variance (ANOVA) followed by Dunnett and Bonferroni post-hoc tests. Differences at p< 0.05 were considered significant.

## Results

**Body weight**

The effect of 28-day oral administration of methanol leaf extract of *D. saxatilis* on animal weight is shown in Table 1. In the control group, significant increase in animal weights at all weeks compared to week zero was observed at p*<*0.05. In group 2 (500 mg/kg),significant increase was also observed at the fourth week compared to week zero at p< 0.05. 

**Renal indices**

Analysis of renal indices showed significant reduction (p<0.05) in serum concentrations of sodium, chloride and bicarbonate (Table 2). At the doses of 250 and 500 mg/kg, the level of sodium and chloride reduced significantly, while bicarbonate reduced at the dose of 250 mg/kg.

**Table 1:** Effect of 28-day administration of methanol leaf extract of *D. saxatilis* on average body weight (g)

**Treatment mg/kg**	**Week 0**	**Week 1**	**Week 2**	**Week 3**	**Week 4**
**Distilled water**	119.00±7.04	134.10±6.90^a^	138.60±5.20^a^	146.50±3.20^a^	146.10±3.71^a^
**Extract 250**	115.80±5.66	117.80±6.44	116.30±5.42	119.63±9.05	128.33±4.80
**Extract 500**	116.20±5.08	110.10±3.11	118.10±2.85	127.00±6.83	140.25±5.95^a^
**Extract 1000**	116.60±4.12	111.30±4.32	109.00±10.1	121.67±8.74	123.17±10.6

Data are presented as mean ± SEM, and analyzed using repeated measures ANOVA followed by Bonferroni test.

‘a’ in superscript represent p*<* 0.05. n= 5.

**Table 2:** Effect of 28-day administration of methanol leaf extract of *D. saxatilis* on renal indices

**Electrolytes **	**Control **	**250mg/kg**	**500mg/kg**	**1000mg/kg**
**Urea (mmol/l)**	10.38±1.35	15.46 ± 2.66	14.70 ± 1.42	15.80 ± 2.68
**Creatinine(μmol/l)**	47.69 ± 6.06	65.05 ± 5.04	59.59 ± 2.59	61.13 ± 4.54
**Chloride (mmol/l)**	122.60±4.06	105.00 ± 4.56^a^	107.00 ± 4.64^a^	111.75 ± 2.96
**Sodium (mmol/l)**	168.80±7.74	139.00 ± 7.15^a^	141.20 ± 5.98^a^	146.00 ± 4.51
**Potassium(mmol/l)**	5.56±0.27	5.95 ± 0.63	5.50 ± 0.32	5.35 ± 0.18
**Bicarbonate (mmol/l)**	28.80±2.25	18.50 ± 1.56^a^	20.80 ± 1.63	21.00 ± 1.16

Data are presented as mean ± SEM, and analyzed using one way ANOVA followed by Dunnett *t-test*.

‘a’ in superscript represent p< 0.05. n = 5.

**Histopathology**

**Figure 1:**
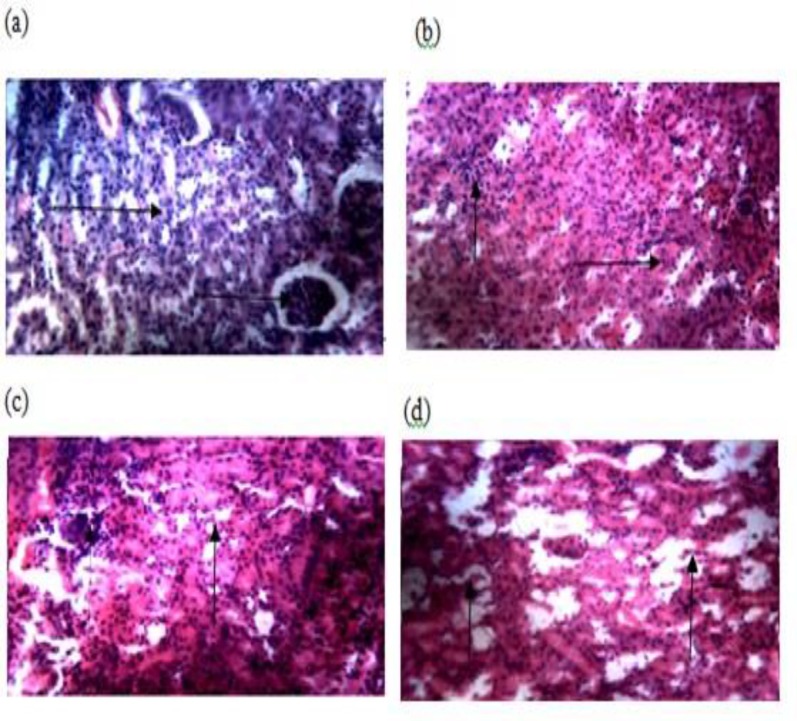
Effect of methanol leaf extract of *D. saxatilis*, representative photomicrographs of the rat kidney in experimental groups; (250x). Control (a), 250 mg/kg (b), 500 mg/kg (c), and 1000 mg/kg of the extract (d

Histopathological examination of the kidney revealed massive necrosis of the glomerulus with tubular distortion and lymphocyte hyperplasia at 250 and 500 mg/kg while intense glomerular and tubular necrosis was observed at 1000 mg/kg of the extract (Figure 1).

## Discussion

Kidney disease or damage may result in accumulation of nitrogenous waste products such as urea and creatinine, and abnormalities of sodium, potassium, chloride, and bicarbonate balance, as these electrolytes helps the body to maintain healthy water balance and stabilize its acid level (Briggs et al., 1996 ▶). Large amounts of circulating toxicants reach the kidneys quickly. The kidneys have high oxygen and nutrient demands because of their workload and they filter one-third of the plasma reaching them and reabsorb high amount of salt and water. As they are reabsorbed, salt concentrates in the kidneys. Changes in kidney pH may increase passive diffusion and cellular concentrations of toxicants, while active secretion processes may concentrate toxicants (Goldstein and Schnellman, 1996 ▶). Creatinine is transported in the blood and cleared through the kidneys; high levels of creatinine in the blood can be a good indicator of kidney failure, hence, kidney failure can greatly affect creatinine clearance (Briggs et al., 1996 ▶). Electrolytes, molecules that are electrically charged, help to move nutrients into and waste products out of the cells. They maintain healthy water balance and help to stabilize the body’s acid level. Low sodium levels are as a result of kidney and adrenal diseases, diuretics, and at times conditions that cause fluid to build up in the body (Halperin and Goldstein, 1994 ▶; Briggs et al., 1996 ▶). The balance of sodium, chloride, and bicarbonate in the blood is a good indicator of how well the kidneys and heart are functioning. Chloride levels fluctuate with sodium levels low chloride levels can occur as a result of chronic lung disease, prolonged vomiting, and metabolic alkalosis. Change in serum chloride indicates an alteration in fluid status and/or acid-base balance (Koch and Taylor, 1992 ▶). Acid-base balance is partly regulated by renal production and excretion of bicarbonate ions. Carbon dioxide -in the form of bicarbonate- is excreted and reabsorbed by the kidneys. High or low bicarbonate levels may signify acid/base or electrolyte imbalance often due to either dehydration or drinking too much water. The primary regulators of bicarbonate are the proximal tubules (Halperin and Goldstein, 1994 ▶). Changes in these electrolytes concentration may be related to the tubular and glomerular necrosis observed in the histopathology of the kidney.
